# Aortic dissection in pregnancy in England: an incidence study using linked national databases

**DOI:** 10.1136/bmjopen-2015-008318

**Published:** 2015-08-20

**Authors:** Amitava Banerjee, Irena Begaj, Sara Thorne

**Affiliations:** 1 University of Birmingham Centre for Cardiovascular Sciences, Birmingham, UK; 2 Quality Outcomes Research Unit, Queen Elizabeth Hospital, Birmingham, UK; 3 Department of Cardiology, Queen Elizabeth Hospital, Birmingham, UK

## Abstract

**Objectives:**

To conduct the first population-level incidence study of aortic dissection in pregnancy using linked hospital-based data in England.

**Setting:**

Hospital-based data (Hospital Episode Statistics (HES) linked with mortality data from the Office of National Statistics), national enquiries (Confidential Enquiries into Maternal Mortality) and surveys (UK Obstetric Surveillance System; UKOSS) of aortic dissection in pregnancy from 2003 to 2011 in England.

**Participants:**

Between 2003 and 2011, all female patients admitted with diagnoses of aortic dissection (not necessarily as the primary cause of admission) and of pregnancy, childbirth and puerperium, were included.

**Outcome measures:**

Diagnosis of aortic dissection during pregnancy, operated or not operated, with outcome of death or live patient from 2003 to 2011 in England.

**Results:**

There were significant differences in characteristics of databases with respect to study population, time of study, recorded event and follow-up of outcomes. On the basis of HES, annual incidence of aortic dissection was 1.23 (95% CI 1.22 to 1.24) per 100 000 maternities. Incidence of aortic dissection with death within 1 year was 0.30 (0.29 to 0.31) per 100 000 maternities. Incidence of aortic dissection increased from 0.74 (0.73 to 0.75) per 100 000 maternities in 2003–2005 to 1.52 (1.51 to 1.53) per 100 000 maternities in 2009–2011. In the Confidential Enquiries into Maternal Deaths, incidence of deaths was highest for 2003–2005 (0.43/100 000 maternities) and lowest for 1997–1999 (0.21/100 000 maternities). In the UK Obstetric Surveillance System, national incidence of aortic dissection was 0.80 (0.50 to 1.50) per 100 000 maternities between 2009 and 2011.

**Conclusions:**

The case of aortic dissection in pregnancy illustrates data limitations regarding complications in pregnancy from different sources in the UK, even for a diagnosis with seemingly few alternative coding and diagnostic possibilities. These limitations should be acknowledged when estimating incidence and outcome.

**Strengths and limitations of this study**

- This is the first analysis to consider aortic dissection in pregnancy across England using data from multiple sources.
- These are the first data regarding the incidence of all aortic dissections (not just deaths) in England for the time period 2003–2011.
- There is considerable variation in the characteristics of different databases of maternal mortality and morbidity, and their findings.
- This study shows that a combination of data sources is probably necessary in order to make optimal estimates of incidence and outcome of aortic dissection in pregnancy, and although routinely collected clinical data may have important uses, there are still significant concerns such as the quality of data linkage.

## Introduction

Aortic dissection, though rare, is an often fatal event.^1^ A recent population-based study from Oxford showed that women have higher mortality from aortic dissection and are more likely to die before hospital assessment,^2^ which was also shown by the world's largest registry of aortic dissection.^3^ Importantly, most individuals with aortic dissection had inadequately controlled hypertension, suggesting that modifiable risk factors may play a role in prevention.^2^ Moreover, women have worse outcomes following surgery for aortic dissection,^3^ and the surgical risk is even higher during pregnancy.^4^ ^5^ The majority of aortic dissections in women of childbearing age occur during pregnancy and have adverse consequences for the mother and the fetus.^6^ Data from the Swedish National Birth Registry in women <40 years of age have shown that pregnancy is associated with a 25-fold increased risk of aortic dissection.^6^ The scientific literature regarding aortic dissection and pregnancy is largely made up of case reports and case series, mostly in individuals with connective tissue diseases, from the last 70 years.^7^ ^8^ A literature review of outcomes in pregnant women with acute aortic dissection from 2003 to 2013 included 59 articles and only 75 patients.^9^ Two population-based studies have considered pregnancy and aortic dissection in the European context,^10^ ^11^ suggesting high mortality from aortic dissection in pregnancy.

In the UK, the Confidential Enquiry into Maternal Deaths (CEMD) has historically provided data regarding aortic dissection and other causes of maternal mortality,^12^ and has shown an increase in deaths from cardiovascular disease during pregnancy in recent years. Although the CEMD (which became the Confidential Enquiries into Maternal and Child Health, CEMACH, in 2003,^12^ and is now known as MBRRACE, Mother and Babies: Reducing Risk through Audits and Confidential Enquiries^13^) provides crucial mortality data and compares favourably with surveillance systems in other countries,^12–14^ it is not designed to detect morbidity or burden of disease and there have been concerns regarding the completeness of its data.^15^ As a result, UKOSS (UK Obstetric Surveillance System) has run prospective surveys into the outcomes of rare conditions in pregnancy, for example, pregnancy-related myocardial infarction (MI).^16^ ^17^ The CEMACH 2006–2008 report highlighted 53 cardiac deaths, of which 7 (13.2%) were due to aortic dissection, translating to 0.31 deaths due to aortic dissection per 100 000 maternities.

Studies of MI have highlighted potentially large discrepancies between primary and secondary care databases and disease registries when estimating incidence, and therefore surveys are unlikely to be accurate for less commonly researched conditions such as aortic dissection.^18^ The use of routinely collected clinical data for public health benefit is an important topic of recent debate, involving both population-level (‘big data’) and individual-level (‘small data’) considerations.^19^ Ideally, estimates of incidence should be made at multiple levels in the healthcare system, or at least at the national level, but this has not been previously attempted, to the best of our knowledge, and no population-level study of hospital-based data, to date, has considered aortic dissection in pregnancy in England or in the UK. We conducted the first detailed analysis of England's national hospital-level data linked to mortality statistics in order to characterise incidence and outcome of aortic dissection in pregnancy and to compare with data from the Confidential Enquiries into Maternal Mortality and UKOSS.

## Aims

The present study had two distinct aims:

- To estimate national incidence of aortic dissection in women during pregnancy from hospital-based data linked to mortality statistics.
- To compare estimates of incidence of aortic dissection from hospital data linked to mortality statistics with data from the Confidential Enquiries into Maternal Mortality and UKOSS in order to test the feasibility of use of ‘big data’.

## Methods

### Study population

Between 1 April 2003 and 31 March 2011, all female patients admitted with a diagnosis of aortic dissection International Statistical Classification of Diseases 10th revision (ICD-10:I710, I711, I712), not necessarily as the primary cause of admission, and with a diagnosis of pregnancy, childbirth and puerperium (ICD-10: O00-O99, Z33), were included in the analysis using ICD-10 codes.^20^ In addition, data for aortic dissection operations were extracted using OPCS4 codes:^21^ L18-L21, L273, L274, L283, L284, L221, K26, K66, K33. The same data regarding aortic dissection from CEMD/CEMACH^22^ and UKOSS^23^ were extracted for the time period between 2003 and 2011 from published reports. The number of aortic dissection events, deaths in hospital and at 1 year, and whether the aortic dissection was surgically managed, were recorded, where possible.

### Databases

The Informatics Department of the University Hospitals Birmingham NHS Trust^24^ has access to Hospital Episode Statistics (HES)^25^ for all inpatient admissions in England, and Office of National Statistics (ONS) mortality statistics.^26^ Data linkage between the two data sets is carried out by the Health and Social Care Information Centre (HSCIC). Data from CEMD/CEMACH^22^/UKOSS^21^ were extracted from published reports.

### Outcomes

A maternal death is defined by the WHO as ‘‘the death of a woman while pregnant or within 42 days of termination of pregnancy”.^20^ Mortality within 42 days of birth was used as the reported outcome despite considerable debate regarding the extension of this time period to reflect the effects of pregnancy and childbirth over a longer timeframe,^27^ because it is the most widely reported. The time to surgery was defined as up to 60 days, in order to include both acute and subacute surgery as stipulated by previous studies.^28^ This time period was also chosen to better reflect the operative burden of aortic dissection in pregnancy.

### Data analysis

Absolute numbers of aortic dissection cases for each year were compared for HES/ONS data versus CEMD/CEMACH/UKOSS. The incidence rates per 100 000 maternities and per 100 000 conceptions were calculated for HES/ONS and compared with estimates from UKOSS. A maternity is a pregnancy resulting in the birth of one or more children, including stillbirths and live births. Conceptions data combine information from registrations of births and notifications of legal abortions occurring in England and Wales for women who are usually resident there (but exclude miscarriages or illegal abortions).^29^ ^30^ Annual data regarding maternities and conceptions were obtained from the HSCIC^29^ and ONS,^30^ respectively. A validation study of HES/ONS data was performed by conducting a search at University Hospitals Birmingham NHS Trust, in order to check fidelity of data linkage between ONS and HES for known local cases of pregnancy-related aortic dissection with review of medical notes. In accordance with ONS guidance, small numbers were suppressed in tables and figures in order to preserve the anonymity of the data.

## Results

### Database characteristics

Table 1 highlights the features of the different UK databases relating to maternal morbidity and mortality. CMED and CEMACH studied maternal deaths and, so, did not include absolute numbers or incidence of aortic dissection that did not result in death. The UKOSS report did include numbers of non-fatal aortic dissection. Death was reported at 42 days in the CEMD/CEMACH/UKOSS data sets, whereas the HES data allowed consideration of inpatient mortality and mortality at 1-year. The remit of the CMED/CEMACH/UKOSS publications was the whole of the UK, whereas the HES data only concerned England. CEMD/CEMACH and UKOSS provided more clinical details regarding the patients with aortic dissection, including their presentation (eg, type A or type B aortic dissection) and operative management. CEMD/CEMACH and UKOSS gave reports for triennia beginning in September of the year in question. In contrast, HES data are collected for the UK financial year (1 April until 31 March of a given year). ONS databases reported conceptions for England and Wales, whereas HSCIC reported maternities for England only. It was not possible to extract the total number of pregnancies (ie, the denominator) from the CEMD or UKOSS publications, making detailed incidence calculations challenging.

**Table 1:** UK Databases of maternal mortality and morbidity

	Database					
Database characteristics	Hospital Episode Statistics (HES)	Office of National Statistics (ONS)	UK Obstetric Surveillance System (UKOSS)	Confidential Enquiry into Maternal Deaths (CEMD)	Confidential Enquiries into Maternal and Child Health (CEMACH)	Mother and Babies: Reducing Risk through Audits and Confidential Enquiries (MBRRACE)
Time period	1987–	1996–	2005–	1952–2002	2003–2011	2012–
–Country	England	England and Wales	UK	Initially restricted to England and Wales, it was extended in 1985 to whole of the UK	UK	England, Wales and Scotland; modified arrangements are in place for Northern Ireland
Retrospective/prospective	Prospective	Prospective	Prospective	Retrospective	Retrospective	Retrospective
Voluntary/mandatory	Mandatory	Mandatory	Voluntary report	Voluntary report	Voluntary report	Voluntary report
Deaths	All deaths	All deaths	Maternal deaths	Maternal deaths	Maternal deaths, stillbirths and infant deaths	Maternal deaths, stillbirths and infant deaths
Events other than death	All hospital episodes	Births	Rare disorders during pregnancy	Nil	Nil	Nil
Lead institution	Health and Social Care Information Centre	UK statistics authority, reporting to UK parliament	National Perinatal Epidemiology Unit (NPEU) at Oxford University	Department of Health	Royal College of Obstetricians & Gynaecologists	National Perinatal Epidemiology Unit (NPEU) at Oxford University

### HES/ONS data

**Figure 1:**
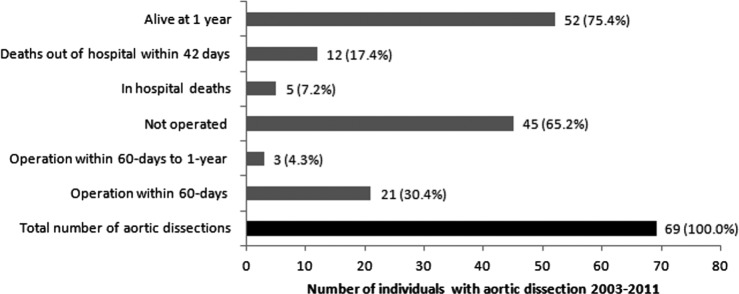
Hospital Episode Statistics/Office of National Statistics data for outcomes of aortic dissection from 2003 to 2011.

According to HES/ONS data, 30 cases of pregnancy-related aortic dissection were identified for the time period 2009–2011, and 69 were identified from 2003 to 2011 (table 2). For 2009–2011, eight cases of AD resulted in operative management within 60 days, and for 2003–2011, 21 underwent operations. From 2003 until 2011, there were 5 in-hospital deaths, 17 deaths within 42 days and 17 deaths at 1 year. The mean age of women with AD during pregnancy did not change significantly over the study period and was 30 years overall. The absolute number of aortic dissection was highest for 2009–2011 (n=30). Inpatient mortality was 7.2%, and mortality was 24.6% at 42 days and 1 year according to the HES data. Operative rates at 60 days and 1 year were 30.4% and 34.8%, respectively (figure 1). Of the 17 deaths in the study period, 13 were recorded in ONS alone and not in HES data.

**Table 2:** Aortic dissection in pregnancy in England 2003–2011 from HES/ONS data

Year	2003–2005	2006–2008	2009–2011	Total
Number of patients	13	26	30	69
Average age	29	31	31	30
Patient operated within 60 days of AD admission	0	13 (50)	8 (26.7)	21 (30.4)
Patient operated 60 days to 1-year of AD admission	*	0 (0)	*	3 (4.3)
In hospital deaths	*	*	*	5 (7.2)
Deaths within 42 days	8 (61.5)	6 (23.1)	*	17 (24.6)
Deaths 42 days to 1 year	0 (0)	0 (0)	*	0 (0)
Aortic dissection recorded in ONS only	7 (53.8)	*	*	13 (18.8)

Numbers are expressed as n (%). An asterisk (*) indicates that the total number of events was <5, and therefore must be suppressed in line with guidance for data governance and anonymity from the ONS.

HES, Hospital Episode Statistics; ONS, Office of National Statistics.

On the basis of the HES data, the overall annual incidence of aortic dissection was 1.23 (95% CI 1.22 to 1.24) per 100 000 maternities and 0.92 (0.91 to 0.93) per 100 000 conceptions. The overall incidence of aortic dissection with death within 1 year was 0.30 (0.29 to 0.31) per 100 000 maternities. Incidence of aortic dissection increased from 0.74 (0.73 to 0.75) per 100 000 maternities in 2003–2005 to 1.52 (1.51 to 1.53) per 100 000 maternities in 2009–2011 (table 3).

**Table 3:** Aortic dissection in pregnancy: incidence and mortality from Hospital Episode Statistics/Office of National Statistics

	Incidence per 100 000/year			
Year	2003–2005	2006–2008	2009–2011	Total
Aortic dissection (maternities)	0.74 (0.73 to 0.75)	1.38 (1.37 to 1.39)	1.52 (1.51 to 1.53)	1.23 (1.22 to 1.24)
Aortic dissection (conceptions)	0.55 (0.54 to 0.56)	1.03 (1.02 to 1.04)	1.16 (1.15 to 1.17)	0.92 (0.91 to 0.93)
Death within 42 days of aortic dissection (maternities)	0.46 (0.45 to 0.47)	0.32 (0.31 to 0.33)	0.15 (0.14 to 0.16)	0.30 (0.29 to 0.31)
Death within 1 year of aortic dissection (maternities)	0.46 (0.45 to 0.47)	0.32 (0.31 to 0.33)	0.15 (0.14 to 0.16)	0.30 (0.29 to 0.31)

### CEMACH/UKOSS data

**Figure 2:**
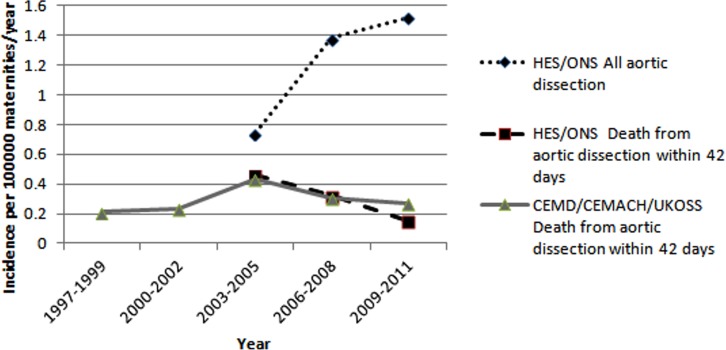
Comparison of incidence of pregnancy-related aortic dissection by different databases (CEMACH, Confidential Enquiries into Maternal and Child Health; CEMDs, Confidential Enquiry into Maternal Deaths; HES, Hospital Episode Statistics; ONS, Office of National Statistics; UKOSS, UK Obstetric Surveillance System).

In the CEMD/CEMACH data, only the total number of aortic dissection deaths was reported in 1997–1999, whereas reports in the following years reported total number of deaths and incidence. The incidence was calculated for 1997–1999 using data regarding number of pregnancies from ONS. Incidence of deaths was highest for 2003–2005 (0.43/100 000 maternities) and lowest for 1997–1999 (0.21/100 000 maternities). The incidence of deaths from aortic dissection has remained stable in 2003–2011 (figure 2). Estimates of incidence for deaths from aortic dissection were similar from HES/ONS and CEMD/CEMACH/UKOSS (figure 2).

Age was only reported in the 2006–2008 report as a median age (34 years). According to the UKOSS, between September 2009 and September 2011, the estimated national incidence of aortic dissection was 0.80 (0.50 to 1.50) per 100 000 maternities, with 12 confirmed cases of aortic dissection in pregnancy. The mean age of women with the disease was 37 years. Three women were managed conservatively while five received an aortic root replacement. The UKOSS ascertained four deaths and eight survivors (case fatality 33%, 95% CI 10% to 65%; table 4). There are no further studies of aortic dissection planned, according to the UKOSS.

**Table 4:** Aortic dissection in pregnancy: data from CEMD/CEMACH/UKOSS

Year	1997–1999	2000–2002	2003–2005	2006–2008	2009–2011	Total
Total	–	–	–	–	12	
Age	–	–	–	34 (median)	37 (mean)	
Deaths within 42 days	5	7	9	7	4	27
Number operated	–	–	–	2	5	
Incidence of AD per 100 000 maternities/year	–	–	–	–	0.80 (0.50–1.50)	
Incidence of deaths within 42 days due to AD per 100 000 maternities/year	0.21 (0.20 to 0.22)	0.23 (0.22 to 24)	0.43 (0.42 to 0.44)	0.31 (0.30 to 0.32)	0.27 (0.17 to 0.50)	

CEMACH, Confidential Enquiries into Maternal and Child Health; CEMD, Confidential Enquiry into Maternal Deaths; UKOSS, UK Obstetric Surveillance System.

In UKOSS 2009–2011, there were seven cases of type A aortic dissection and three of type B aortic dissection using Stanford criteria. Only one case was reported in association with Marfan's disease; one woman had pre-existing aortic coarctation and a bicuspid aortic valve. Detailed data regarding the type of aortic dissection and aetiological factors were not routinely reported in the prior reports.

## Discussion

### Aortic dissection

There have been recent concerns about the quality of data regarding maternal mortality in the UK setting.^31^ ^32^ To the best of our knowledge, this is the first analysis to consider aortic dissection in pregnancy across England using data from multiple sources, including HES/ONS. The only comparative data sets are from CEMD, CEMACH and UKOSS, and although the incidence estimates for death from aortic dissection are similar when compared with HES/ONS, we have shown considerable variation in database characteristics and findings of those data. The incidence of aortic dissection is showing an upward trend in recent years while there is a downward trend in mortality from aortic dissection over the same time period, according to HES data. Notably, the incidence estimated from HES/ONS is almost double the recent estimate on the basis of UKOSS (1.52 vs 0.80/100 000 maternities). A study of maternal mortality from 1993 to 2008 estimated the incidence of mortality due to aortic dissection as 0.42/100 000 live births,^10^ which is comparable to our estimates for England from HES/ONS and those for the UK from CEMD/CEMACH/UKOSS.

The Confidential Enquiries into Maternal Deaths did not consider non-fatal events and UKOSS has only considered aortic dissection in one report; there is no further report for AD planned. In order to conduct disease monitoring of any condition, particularly mortality and morbidity experienced during pregnancy, there is an urgent need for ongoing surveillance in order to map trends and highlight health service needs. It is not sufficient to only monitor trends in mortality, and any surveillance programme must be consistent in its recording and in its reporting. National data sets offer advantages over surveys and audits of mortality for these purposes, and are currently-underused.

### Big data

HES/ONS data and the CEMD/CEMACH/UKOSS data both have significant limitations with regard to aortic dissection in pregnancy. This is surprising and disappointing for a ‘red-flag’ diagnosis such as aortic dissection in pregnancy, which should not have as many incorrect alternative codes/diagnoses during hospital admissions or in mortality data. For example, for HES/ONS data, 13/17 deaths were only coded in ONS and would have been missed by HES data alone. It is important to understand the patient pathway and the levels in the health system and chronology of coding in order to interpret these data. The higher number of deaths in ONS compared with HES is likely to be due to the fact that a significant number of deaths from aortic dissection are out-of-hospital and would therefore not be recorded in ONS. In addition, hospital deaths that occur within 24 h of admission or as a result of an operation would ordinarily be investigated by postmortem, the results of which would not be recorded in HES.

The mortality rate from HES/ONS data was 24.6% for aortic dissection in pregnancy, which is similar to the published literature (21% for type A vs 23% for type B dissections).^9^ An anomaly in our data is the relatively low operative rate in aortic dissection in pregnancy (34.8% at 1 year). A recent literature review suggested that type A dissections were the most common form of aortic dissection in pregnancy, accounting for 77% of all cases,^9^ and the vast majority of these cases would be expected to result in surgical management. Therefore, there is likely to be a high proportion of miscoding or ‘missed coding’ of operative management of aortic dissection in pregnancy in HES/ONS. National databases such as those for MI, coordinated by the National Institute for Cardiovascular Outcomes Research (NICOR), may have helped in explaining the high rates of unoperated aortic dissection, but no relevant database currently exists for pregnancy. Moreover, using currently available routinely collected clinical data, analysis of aetiology (eg, hypertension, diabetes) or presentation (eg, type A or type B) is not possible. Such data are required not only to better understand the disease process but also to improve prevention and management.

Although CEMD/CEMACH/UKOSS data are for the UK, whereas HES/ONS is only for England, the number of deaths in the former data set seems to be too low for aortic dissection in pregnancy. There are no coordinated primary care data regarding aortic dissection in pregnancy with which to compare our results. We used linkage of HES data with ONS and there seems to be a significant discrepancy between HES and CEMD/CEMACH/UKOSS for this particular patient population. The incidence and the case fatality calculated are affected greatly by the choice of data set, the denominator (maternities vs conceptions) and the event in question (cases or deaths). These issues are directly relevant to incidence estimates for any disease.

### Limitations

This study is limited by the data sets used. For HES/ONS data, no information is available regarding primary care and no detail other than coding could be obtained. The HES data are retrospective. For CEMD/CEMACH/UKOSS, we had access to full reports but not to data. UKOSS relies on hospitals to submit monthly surveys and although response rates are high (>85%), the surveys often have incomplete data. UKOSS states that “Extensive work to date, including through various professional societies and the Intensive Care National Audit and Research Centre database, does not indicate significant under-ascertainment of cases.” However, for 2009–2011, 85% of maternal deaths have been identified although complete information is only available for about 52%.^16^ It is difficult to directly compare CEMD/CEMACH/UKOSS and HES/ONS due to the differential study populations (UK vs England) and the other variations in data characteristics. For example, the former studies report data regarding triennia whereas the latter can be used to produce annualised rates. Neither CEMD/CEMACH/UKOSS nor HES/ONS are able to comment on fetal outcomes. In general, the available databases do not provide much information other than diagnosis, hence details of the site and type of aortic dissection, whether dissection occurred during or after pregnancy and proportion of live births after surgery, are unknown.

## Conclusions

The case of aortic dissection in pregnancy illustrates limitations of data regarding complications in pregnancy from different sources in the UK setting, even for a diagnosis with seemingly few alternative coding and diagnostic possibilities. These limitations should always be acknowledged when making estimates of incidence and outcome. A recent study comparing the accuracy of diagnosis of MI from primary care, HES and disease registries not only showed that there were discrepancies across data sets, but also that increasing the number of linked data sets increased the ‘pick-up’ rate considerably.^18^ These issues are pertinent as the UK grapples with how best to manage ‘Big Data’ for healthcare.^33^ Interestingly, the quality of surveillance of causes of global maternal mortality has greatly improved through the Global Burden of Disease Study, and the UK must keep up with this trend for its national data.^34^

A combination of data sources is probably necessary in order to make optimal estimates of incidence and outcome of aortic dissection in pregnancy, and although routinely collected clinical data may have important uses, there are still significant concerns such as the quality of data linkage. Across data sets, standardisation of minimal data collection will reduce data heterogeneity and missing data. It is of concern that there are no further planned studies of aortic dissection in pregnancy, according to the UKOSS. Prospective population-based studies and registries may still offer important disease-specific information, but incidence and outcome can be estimated from large data sets of routinely collected clinical data in the UK.
